# Ureteral Metastasis From Prostate Cancer: A Report of Two Cases

**DOI:** 10.7759/cureus.62676

**Published:** 2024-06-19

**Authors:** Renish N Contractor, Matthew D McGee, Prathap Simhadri, Cameron Gerhold, Evan Fynes

**Affiliations:** 1 Urology, Florida State University College of Medicine, Daytona Beach, USA; 2 Internal Medicine, Lake Erie College of Osteopathic Medicine, Bradenton, USA; 3 Internal Medicine/Nephrology, Florida State University College of Medicine, Daytona Beach, USA; 4 Orthopedic Surgery, Florida State University College of Medicine, Daytona Beach, USA; 5 Urology, Advanced Urology Institute/Advent Health, Daytona Beach, USA

**Keywords:** ureter, lower urinary tract, hydronephrosis, ureteral metastasis, prostate adenocarcinoma

## Abstract

Prostate adenocarcinoma (PCa) is the second most common cause of cancer in men, but metastases to the ureter are exceedingly rare. Here, we present two cases with differing clinical symptoms and treatment courses but ultimately the same diagnosis. The two cases presented here had differing clinical presentations: one with lower urinary tract symptoms and the other with hydronephrosis. Systemic therapy with a luteinizing hormone-releasing hormone (LHrH) agonist appears to help with clinical outcomes in both cases reported here. Although such cases are extremely rare, consideration as a differential and early detection can impact a patient's clinical outcomes. For patients with PCa that present with obstructive urinary symptoms, there may be a clinical benefit to perform a thorough metastatic work-up for seeding to other parts of the urinary tract.

## Introduction

Prostate adenocarcinoma (PCa) is the second leading common cause of cancer in men worldwide, with an incidence of 25.3 per 100,000. With the development of screening guidelines to help with the early detection of disease, the incidence of PCa has been on the rise [1,2]. The majority of the metastasis from PCa is to the regional lymph nodes, bone, lungs, distal lymph nodes, and liver. In such cases, metastatic disease results due to hematogenous spread. Skeletal metastasis is most commonly found, while metastasis to the renal pelvis and proximal ureter is exceedingly rare [3]. In cases of metastases to the ureter, it is not known whether this results from lymphatic or hematogenous spread [4]. The most common causes of ureteral metastasis are from breast, gastric, and colorectal cancer [5]. In patients diagnosed with PCa, approximately 20% of cases develop metastatic disease [6]. Distant spread correlates with a poor prognosis, with a five-year relative survival rate of only 29.8% [7]. Per the literature as of 2015, there have been only 46 reported cases of prostatic malignancy with ureteral metastasis. This underscores the urgent need for reporting, as the scarcity of research on this matter directly influences treatment strategies [8]. Here, we report two cases from 2021 to 2022 and 2023 to 2024 of primary prostatic adenocarcinoma with metastasis to the ureters, presenting with unilateral ureteral obstruction.

## Case presentation

Case I 

**Figure 1:**
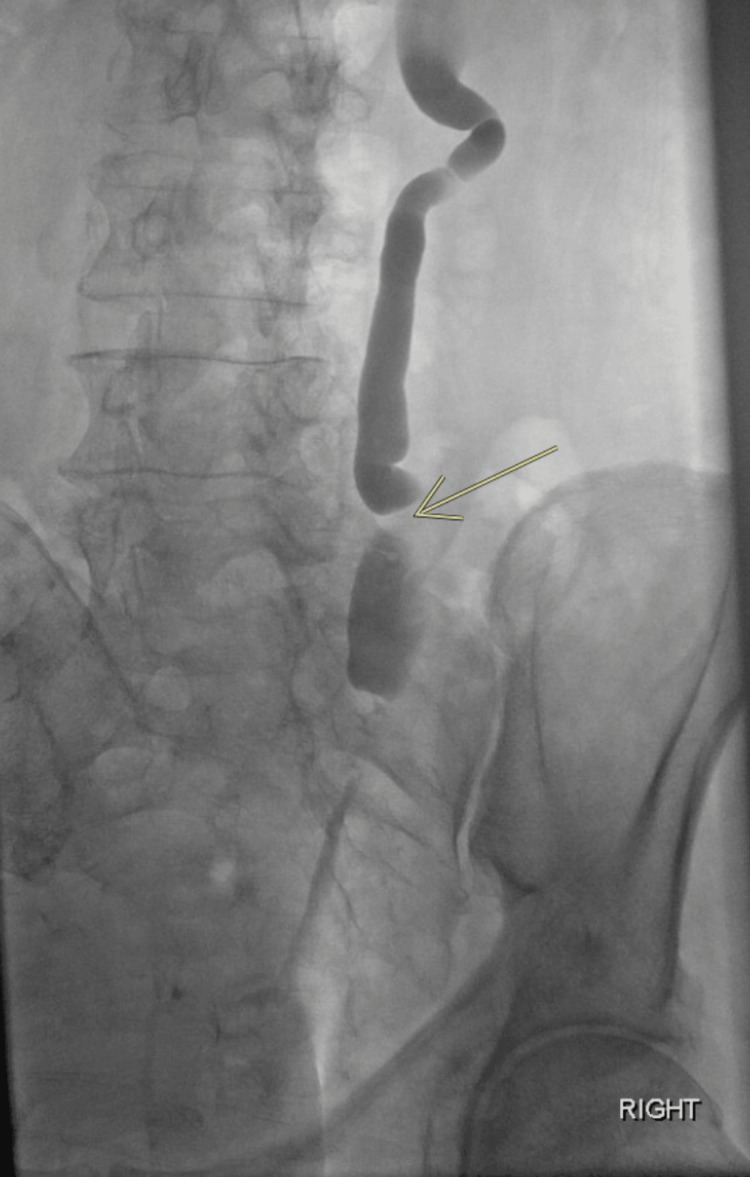
Right antegrade nephrostogram showing shouldered margin with severe upstream hydroureter, considered pathognomonic for a malignant ureteral obstruction The arrow points to a 5 cm filling defect in the distal right ureter, which is consistent with urothelial cell carcinoma

A 78-year-old male with chronic kidney disease (CKD), stage IV, and benign prostatic hyperplasia (BPH) was seen by his nephrologist for a renal ultrasound, showing mild to moderate right hydronephrosis. The patient was referred to urology for further evaluation. He was found to have an International Prostate Symptom Score (IPSS) score of 11 and was prescribed tamsulosin. His prostate-specific antigen (PSA) was 3.4 ng/mL, and computed tomography (CT) of the abdomen and pelvis without contrast showed right hydronephrosis with an abrupt change in the right distal ureter. The urine cytology was negative, and the patient underwent a cystoscopy with an attempted right retrograde pyelogram, which was unable to visualize the right ureteral orifice due to his enlarged prostate. A right nephrostomy tube was placed by interventional radiology, and an antegrade nephrostogram was performed showing a 5 cm filling defect in the distal right ureter which was consistent with urothelial cell carcinoma (Figure 1). The patient was subsequently referred to another urologist who performed a right robotic nephroureterectomy, with pathology coming back as poorly differentiated adenocarcinoma, likely prostatic in origin. His PSA was rechecked, coming back at 5.5 ng/mL. A transrectal ultrasound with prostate biopsy was performed, showing 12/12 cores with Gleason 9 (4+5) PCa. Further chest X-ray, bone scan, and CT abdomen and pelvis were negative for metastatic disease. The patient was started on a luteinizing hormone-releasing hormone (LHrH) agonist, abiraterone, and prednisone daily. His PSA has been <0.04 ng/mL for the past two years along with stable renal function.

Case II 

**Figure 2:**
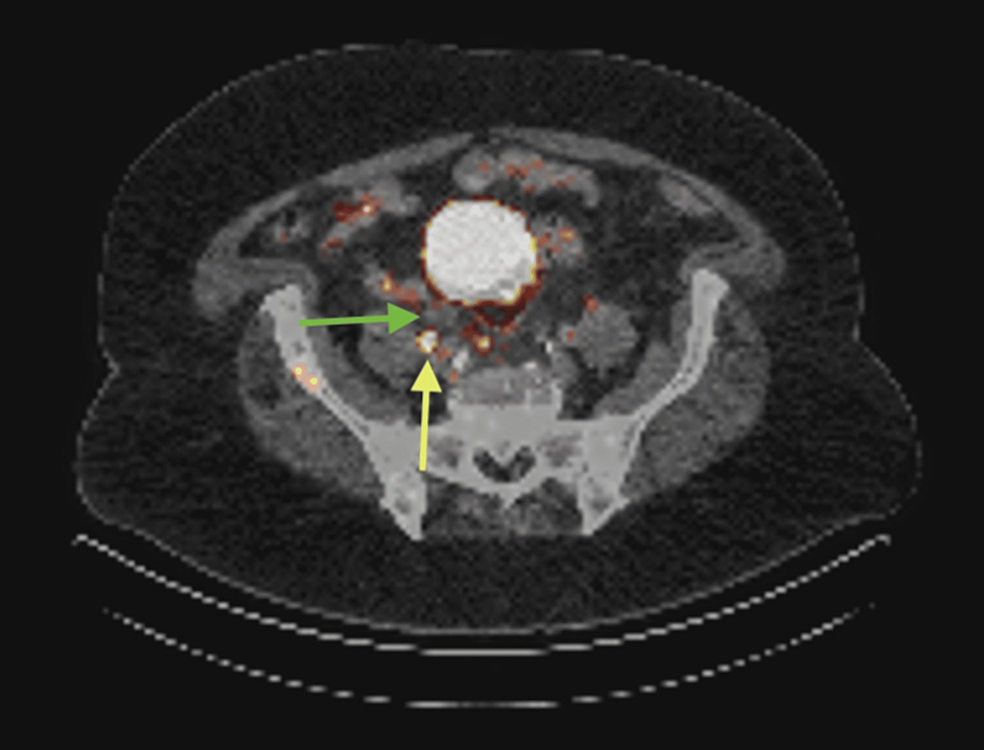
Case II prostate-specific membrane antigen (PSMA) positron emission tomography (PET) scan The yellow arrow here shows metastatic adenopathy in the retroperitoneum and pelvis. The green arrow highlights right hydroureteronephrosis to the distal ureter

**Figure 3:**
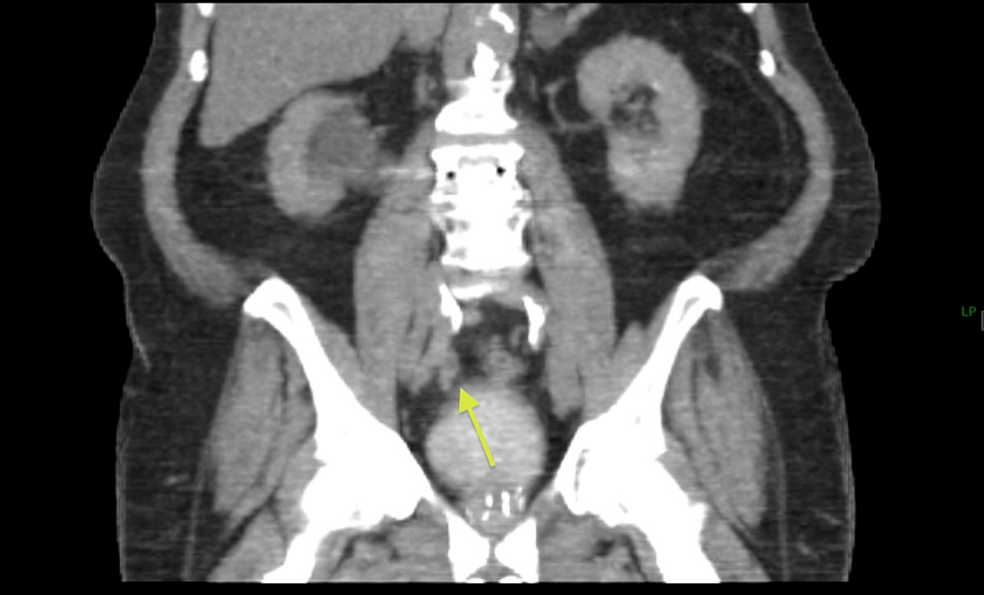
Case II CT urogram The arrow here shows narrowing in the distal right ureter with proximal hydroureteronephrosis

A 75-year-old male presented with a history of BPH with bothersome lower urinary tract symptoms. His symptoms were controlled with tamsulosin, finasteride, and mirabegron. His previous PSA in April 2022 was 3.85 ng/mL (or 7.7 ng/mL corrected with finasteride). His previous urologist had passed away, and this was his initial visit. At this time, his PSA increased to 15.4 ng/mL (or 30.8 ng/mL corrected with finasteride). A confirmatory PSA was performed which was also elevated at 18.1 ng/mL (or 36.2 ng/mL corrected with finasteride). Subsequently, an MRI of the prostate revealed a size of 42 g of the prostate gland. Additionally, the MRI showed a Prostate Imaging Reporting and Data System (PI-RADS) 5 lesion involving almost the entirety of the prostate, including both peripheral and transition zones, with suggestion of bilateral seminal vesicle (SV) invasion. The MRI also revealed a 2 cm bone lesion in the right acetabulum, multiple enlarged lymph nodes in the pelvis, and a dilated distal right ureter with central filling defect. An MRI-guided biopsy was then performed, which revealed that nine out of twelve cores with all of each core had Gleason 9 (4+5) PCa, which meant that the patient had a high grade malignancy. A follow-up prostate-specific membrane antigen (PSMA) positron emission tomography (PET) scan showed metastatic adenopathy in the retroperitoneum and pelvis and bone lesions in the right anterior acetabulum and right iliac bone. It also showed right hydroureteronephrosis down to the distal ureter (Figure 2). The patient was then started on apalutamide, a type of androgen receptor inhibitor, and an LHrH agonist. Due to the incidental right hydroureteronephrosis found on the PSMA scan, a CT urogram was performed which showed narrowing in the distal right ureter with proximal hydroureteronephrosis (Figure 3). He underwent a cystoscopy, right ureteroscopy, and ureteral biopsy with stent placement. The pathology report of the right ureteral biopsy showed metastatic adenocarcinoma of the prostate. With the apalutamide and LHrH agonist, the patient’s PSA responded and came down to 0.1 ng/mL. He was referred to oncology who deferred care back to urology for a plan of care as they did not believe chemotherapy would have any additional benefit. The patient is currently tolerating the stent well. The current plan is to leave the stent in place and await response to medical therapy, in hopes that the tumor will shrink, relieving the obstruction and subsequently allowing the patient to live stent free.

## Discussion

The two cases described here were both elderly males with a medical history of BPH and prostate cancer. Both patients presented with elevated PSA levels and BPH-related urinary symptoms; however, the clinical course differs among the two. In Case I, the patient has CKD stage IV in addition to BPH and presented with right-sided hydronephrosis which led to further investigations. Initial evaluation included an IPSS score assessment, renal ultrasound, and CT scan. A change in the distal ureter led to the patient undergoing cystoscopy, attempted pyelogram, and nephrostomy tube placement. With pathology showing poorly differentiated adenocarcinoma likely of prostatic origin and a high Gleason score of 9 (4+5), surgical treatment with nephroureterectomy followed by medical management of hormonal-related therapy and steroids (LHrH agonist, abiraterone, prednisone) was given, and the patient has had well-controlled PSA levels (<0.04 ng/mL) for the past two years with stable renal function. Case II developed lower urinary tract symptoms leading to a work-up showing an elevated PSA and high grade malignancy. MRI and PSMA PET scans confirmed metastatic disease, and biopsy showed a Gleason score of 9 (4+5) in all sampled cores. Hormonal-related therapy with apalutamide and an LHrH agonist was given, and stents were placed to relieve ureteral obstruction. The patient’s PSA responded well to treatment, decreasing to 0.1 ng/mL.

In summary, Case I presented with more renal manifestations and underwent surgical intervention with medical therapy, while Case II presented with widespread metastatic disease and underwent medical therapy followed by intervention to relieve urinary obstruction caused by metastatic involvement of the ureter. The different clinical presentations underscore the importance of keeping a broad and vigilant approach to a differential diagnosis. Although one presented with lower urinary symptoms and the other with a development of hydronephrosis, both ultimately revealed metastatic cancer with further work-up.

In the early 20th century, ureteral metastases by PCa were only found incidentally during autopsy. With the increasing use of ultrasound and ureteroscopy, clinical reporting of incidental ureteral metastases has increased. Despite this, metastatic tumors to the ureter are relatively rare and an uncommon cause of ureteric obstruction. Since being first reported in the literature by Stow (1909) there have only been a total of 400 cases, with the majority being from breast or colorectal cancer, and an even smaller number of those being due to PCa metastasis [9,10]. However, only a small percentage of patients with ureteral metastasis are reported as most patients remain asymptomatic, up to 85% [11]. In cases where patients are symptomatic, the most frequently reported symptoms are flank pain and hematuria, although both are relatively rare [12]. In the two cases reported here, hydronephrosis was the presenting symptom found in both patients. In symptomatic cases, the symptoms resemble those found in other renal and ureteral tumors. Diagnosis can be made via ureteroscopy and biopsy [13]. 

The ureters are typically resistant to metastatic disease by hematogenous and lymphatic spread due to their lack of a continuous network of blood vessels and lymphatic vessels. Additionally, lymphatic tumor spread to the ureters is further complicated by a countercurrent effect whereby the flow of lymphatics in the lower ureter is caudal, while the lymphatic flow in the other pelvic organs is cranially. This further decreases the chance of lymphatic metastases to the ureter [14]. Metastases to the ureter can involve any portion of the ureter [15] and can occur in three different ways. Bilateral ureteral involvement is seen in approximately 25% to 70% of cases [16]. The most common type, type I, consists of periureteral adventitial layer involvement by the primary tumor cells. Metastases by type I usually result in compression of the ureteral wall. Type II consists of involvement of a portion of the layers of the ureter or transmural involvement along with tumor cells found in the muscular, perilymphatic, or vascular layer of the ureter. Type III involves the local mucosa of the ureter and may also include the muscularis layer. Metastatic ureteral involvement by type I or II commonly results in stricture formation or obstruction of the ureter. Type III can be visualized radiographically as filling defects within the ureteral lumen [10].

Regardless of the type of metastasis to the ureter, it is imperative that urologists and other physicians can identify this problem early on. The vague presentation of the few known cases of metastatic PCa to the urethra can make this diagnosis a clinical challenge for even the most esteemed urologists. Furthermore, it is important that clinicians do not rule out the possibility of metastatic disease for younger patients, as men under the age of 50 have an increased risk of death within two years of being diagnosed with metastatic PCa [17]. All individuals found to have metastatic PCa to the urethra should be screened to rule out prostatic metastasis to other locations. Increasing awareness of this rare, but deadly, pathology can help physicians detect this issue earlier, improving the survivability of this disease process and improving the quality of life of affected individuals.

## Conclusions

In this paper, we report two cases of prostate cancer metastasizing to the ureter. Although PCa is the second most common cause of cancer in men worldwide, metastasis to the ureter is exceedingly rare, with very few cases reported in history. The two cases presented here had differing clinical presentations: one with lower urinary tract symptoms and the other with hydronephrosis. Although such cases are extremely rare, consideration as a differential and early detection can impact a patient's clinical outcomes. For patients with PCa that present with obstructive urinary symptoms, there may be a clinical benefit to screen for seeding to other parts of the urinary tract.
